# A detailed methodology to model the Non Contact Tonometry: a Fluid Structure Interaction study

**DOI:** 10.3389/fbioe.2022.981665

**Published:** 2022-10-04

**Authors:** Elena Redaelli, Jorge Grasa, Begoña Calvo, Jose Felix Rodriguez Matas, Giulia Luraghi

**Affiliations:** ^1^ Aragón Institute of Engineering Research (I3A), University of Zaragoza, Zaragoza, Spain; ^2^ Centro de Investigación Biomecánica en Red en Bioingenieria, Biomateriales y Nanomedicina (CIBER-BBN), Zaragoza, Spain; ^3^ LaBS, Department of Chemistry, Materials and Chemical Engineering “Giulio Natta”, Politecnico di Milano, Milan, Italy

**Keywords:** corneal biomechanics, numerical modeling, fluid-structure interaction (FSI), non-contact tonometry, intraocular pressure (IOP)

## Abstract

Understanding the corneal mechanical properties has great importance in the study of corneal pathologies and the prediction of refractive surgery outcomes. Non-Contact Tonometry (NCT) is a non-invasive diagnostic tool intended to characterize the corneal tissue response *in vivo* by applying a defined air-pulse. The biomarkers inferred from this test can only be considered as indicators of the global biomechanical behaviour rather than the intrinsic biomechanical properties of the corneal tissue. A possibility to isolate the mechanical response of the corneal tissue is the use of an inverse finite element method, which is based on accurate and reliable modelling. Since a detailed methodology is still missing in the literature, this paper aims to construct a high-fidelity finite-element model of an idealized 3D eye for *in silico* NCT. A fluid-structure interaction (FSI) simulation is developed to virtually apply a defined air-pulse to a 3D idealized eye model comprising cornea, limbus, sclera, lens and humors. Then, a sensitivity analysis is performed to examine the influence of the intraocular pressure (IOP) and the structural material parameters on three biomarkers associated with corneal deformation. The analysis reveals the requirements for the *in silico* study linked to the correct reproduction of three main aspects: the air pressure over the cornea, the biomechanical properties of the tissues, and the IOP. The adoption of an FSI simulation is crucial to capture the correct air pressure profile over the cornea as a consequence of the air-jet. Regarding the parts of the eye, an anisotropic material should be used for the cornea. An important component is the sclera: the stiffer the sclera, the lower the corneal deformation due to the air-puff. Finally, the fluid-like behavior of the humors should be considered in order to account for the correct variation of the IOP during the test which will, otherwise, remain constant. The development of a strong FSI tool amenable to model coupled structures and fluids provides the basis to find the biomechanical properties of the corneal tissue *in vivo*.

## 1 Introduction

The cornea is the primary refractive surface of the eye, owning 75% of its refractive power (Chong and Dupps Jr, 2021). This optics capability is highly correlated to its mechanical properties (Ávila et al., 2021), therefore understanding the corneal mechanical behaviour is crucial for the study of some ocular pathologies and the prediction of refractive surgery outcomes (Kling and Hafezi, 2017). Non-Contact Tonometry (NCT) is a non-invasive diagnostic tool intended to characterize the corneal response *in vivo* by applying a defined air-puff. During the test, the cornea deforms inward and then recovers to its original shape, hence it goes through three main phases: first applanation point, highest concavity point and second applanation point. In the Corvis ST^®^ (Corvis, Oculus Optikgeräte GmbH, Wetzlar, Germany) (Hong et al., 2013) tonometer, the dynamic deformation of the equatorial plane of the cornea is recorded through a high-speed camera, 140 horizontal 8 mm frames are taken over a period of 33 mas (Esporcatte et al., 2020). Based on the images, biomarkers such as the deflection amplitude and the peak distance are calculated. These parameters can only be considered as indicators of the mechanical behaviour rather than the intrinsic mechanical properties of the corneal tissue. Indeed, the response of the cornea to the air-puff depends on the combination of four factors: the intraocular pressure (IOP), the thickness of the cornea, the external loads, and the mechanical properties of the cornea and the surrounding tissue (Ariza-Gracia et al., 2015). Since only the mechanical properties of the cornea and surrounding tissue are unknown, it is possible to construct an inverse finite element method to isolate the influence of each factor in the corneal response and identify the parameters of the mathematical model used to reproduce the mechanical response of the corneal tissue. The inverse finite element method requires accurate and reliable modelling of the NCT to effectively translate the results of the test into clinical data.

The approaches to model the interaction between the air puff and the structure of the eye can be classified according to the numerical analysis adopted. Initial studies assumed a structural finite element analysis (FEA) technique. In Eliasy et al. (Eliasy et al., 2019) and Rahmati et al. (Rahmati et al., 2021) the eye is the structural part with proper mechanical properties, while the action of the air puff test is simulated by a time-dependent pressure. Montanino et al. (Montanino et al., 2018) (Montanino et al., 2019) employing FEA underlined the need of modelling the filling fluid of the humors, to avoid a wrong estimate of the material properties of the eye tissues. The limitation of FEA modeling is the lack of correlation between the external load and the corneal geometry and mechanical properties. For this reason, other works (Muench et al., 2019) (Nguyen et al., 2019) (Huang et al., 2020) conducted a computational fluid dynamic (CFD) analysis to find the correct pressure profile to be applied to the corneal tissue. The air is modeled as a fluid and the eye as a rigid body. Then, the deformable eye is loaded with the pressure profile obtained from the CFD. In these simulations, the pressure profile depends on the initial geometry of the eye, and is not modulated by the corneal mechanical properties as happens in reality. Hence, the adoption of the fluid-structure interaction (FSI) analysis, where the structural domain is combined with the fluid domain, has become of great interest. Ariza-Gracia et al. (Ariza-Gracia et al., 2018), Maklad et al. (Maklad et al., 2020b) and Issarti et al. (Issarti et al., 2021) demonstrated that the best numerical approach to reproduce the NCT is the FSI simulation. Ariza-Gracia et al. (Ariza-Gracia et al., 2018) considered a 2D model, which limits the study to an isotropic cornea, Maklad et al. (Maklad et al., 2020b) and Issarti et al. (Issarti et al., 2021) used a limited fluid domain. Even though the cornea contains multi-scale structures with distinct patterns of fiber organization (Blackburn et al., 2019), to the best of authors knowledge, current FSI solutions (Ariza-Gracia et al., 2018) (Maklad et al., 2020b) (Issarti et al., 2021) do not account for the anisotropy of the cornea in the simulation. This work presents a high-fidelity fluid-structure finite-element model of an idealized 3D eye to virtually reproduce the NCT.

## 2 Methods

### 2.1 Structural model of the eye

#### 2.1.1 Geometry

**FIGURE 1:**
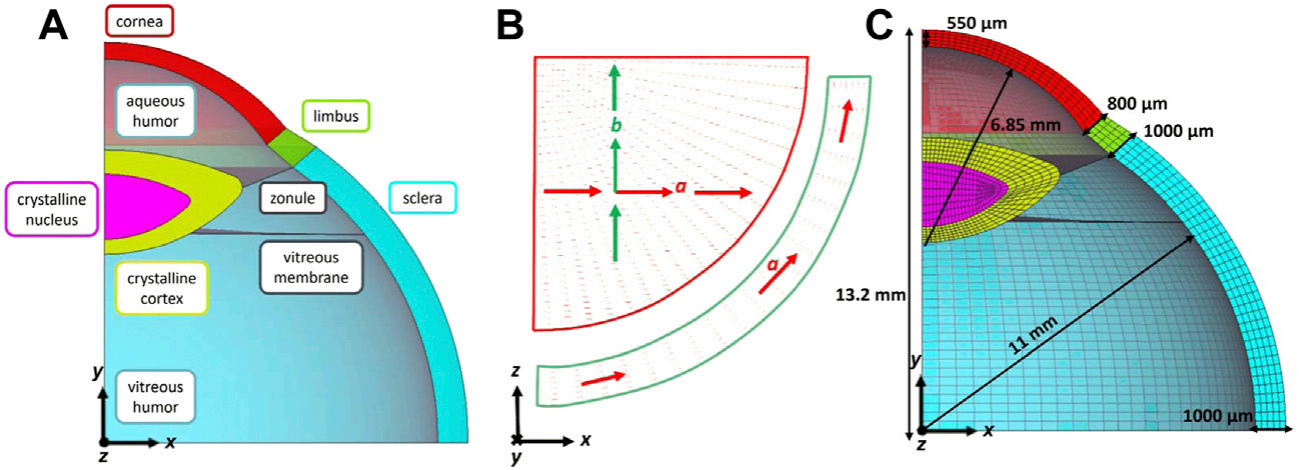
**(A)** Section of the 3D structural parts: cornea, limbus, sclera, crystalline lens nucleus, and cortex, zonule, vitreous membrane, vitreous and aqueous humors; **(B)** local reference systems defined in each element of the cornea and limbus to model two family of collagen fibres **(A,B)** in the corneal plane and one family of fibres **(A)** in the tangential direction of the limbus; **(C)** Hexahedral mesh with five elements in the thickness.

A 3D model of an eye (Figure 1A) is constructed based on averaged anatomic measures taken from literature (Ariza-Gracia et al., 2018) (Cabeza-Gil et al., 2021). It contains cornea, limbus, sclera, crystalline lens differentiated in cortex and nucleus, aqueous and vitreous humors separated by the vitreous membrane, and ciliary zonule modelled as a thin membrane. Due to the double symmetry only a quarter of a middle eye has been considered and symmetric boundary conditions are imposed, rigid body motions are prevented in the bottom and lateral surfaces. The eye is meshed with hexahedral solid elements (Figure 1C) with full integration except for the vitreous membrane and the ciliary zonule meshed with quadratic shell elements with full integration (further discretization details are in the following section). The mesh is realized through the commercially available software ANSA Pre Processor v22.01 (BETA CAE Systems, Switzerland).

### 2.1.2 Constitutive material models and zero pressure configuration

The cornea and the limbus are modelled as anisotropic nearly-incompressible hyperelastic materials to account for the influence of the collagen fibres present in the tissues. In the cornea, two families of mutually perpendicular collagen fibres tangent to the corneal surface (Meek and Knupp, 2015) have been considered: 1) a nasal-temporal direction coincident with the global *x*-axis, and 2) a superior-inferior direction coincident with the global *z*-axis, whereas the limbus is composed of one circumferential family of fibres as shown in Figure 1B. The Holzapfel Gasser Ogden strain energy function (Holzapfel et al., 2000) is adopted for the cornea and the limbus

$$\Psi =C_{10}\left({\bar{I}}_{1}-3\right)+\frac{k_{1}}{2k_{2}}\underset{i=4,6}{\sum }e^{k_{2}{\left({\bar{I}}_{i}-1\right)}^{2}}+\frac{1}{k}{\left(J-1\right)}^{2}$$ (1)

where *C*_10_ is the material constant associated with the extracellular matrix behaviour, *k*_1_ is the material constant associated with the fibres stiffness and *k*_2_ with the fibres non linearity. *k* is the bulk modulus, in case of nearly incompressibility the parameter can be thought of as a penalty factor enforcing the incompressibility constraint. ${\bar{I}}_{1}$ is the first invariant, ${\bar{I}}_{4}$ , ${\bar{I}}_{6}$ are respectively the fourth and sixth pseudo-invariants of the modified right Cauchy Green deformation tensor $\left(\bar{C}\right)$ and *J* is the determinant of the deformation gradient (*F*).

$$C=F^{T}\cdot F\;\bar{C}=J^{-2/3}\cdot C$$ (2)

$${\bar{I}}_{1}=tr\left(\bar{C}\right)\;{\bar{I}}_{4}=m\cdot m^{T}\;{\bar{I}}_{6}=n\cdot n^{T}$$ (3)

**m** and **n** are the vectors defining the direction of the fibres.

In this first study the mechanical parameters of cornea and limbus are estimated fitting experimental stress strain data reported in the literature (Huang et al., 2020). The same mechanical properties have been assumed for all families of fibres. To simplify the subsequent sensitivity analysis, the sclera is modelled as an isotropic hyperelastic material with a Neo Hookean strain energy density function in which the stiffness is only defined by one parameter.

$$\Psi =C_{10}\left({\bar{I}}_{1}-3\right)+\frac{1}{k}{\left(J-1\right)}^{2}$$ (4)

*C*_10_ of the sclera is estimated fitting experimental stress strain data reported in the literature (Eilaghi et al., 2010). Since the deformations of the internal tissues are not large, the crystalline, ciliary zonule and vitreous membrane are modelled as linear elastic materials (Kim et al., 2019) (Wilde et al., 2012) (Krag and Andreassen, 2003). Note that, since the capsule is not included in the model, the Young modulus of the crystalline cortex is arbitrarily selected as the average of the Young modulus of the crystalline cortex reported in (Wilde et al., 2012) and the Young modulus of the capsule reported in (Krag and Andreassen, 2003). All the material constants used in the model are reported in Table 1.

**TABLE 1:** Material parameters of the different eye tissues incorporated in the finite element model.

Material properties					
Part	*C*_10_ [MPa]	*k*_1_ [MPa]	*k*_2_ [ − ]	*ρ* [g/*mm* ^3^]	Ref
Cornea	0.05	0.010	100	0.0011	(Huang et al., 2020)
Limbo	0.05	0.010	100	0.0011	(Huang et al., 2020)
Sclera	0.8	–	–	0.0011	(Eilaghi et al., 2010)
**Part**	**E [MPa]**			***ρ*** ** [g/** ***mm*** ^**3**^ **]**	
Crystalline Lens Nucleus	0.0003			0.0011	(Wilde et al., 2012)
Crystalline Lens Cortex	0.35			0.0011	(Wilde et al., 2012) (Krag and Andreassen, 2003)
Ciliary Zonule	0.35			0.0011	(Kim et al., 2019)
Vitreous Membrane	0.35			0.0011	(Kim et al., 2019)

Considerable attention must be paid to the formulation of the humors which are incompressible fluids pressurized at a spatially homogenous intraocular pressure (IOP). The humors are modelled as a cavity in which the relationship between pressure and volume is controlled. Since the initial pressure of the cavities is zero, a positive input flow rate is imposed until the target IOP of 15 mmHg is reached. Then, the volumes are closed, and the humours behave as incompressible fluids.

Since the average dimensions of the eye (Figure 1C) corresponds to the pressurized configuration, in a first step of the simulation, the zero-pressure configuration of the eye is found through the iterative algorithm described in Ariza Gracia et al. (Ariza-Gracia et al., 2016).

In addition, since a dynamic analysis is used for the structural solver, a mass weighted damping has been adopted for all the parts with a damping constant of 0.1 ms^−1^. The mass weighted damping has the role of damping the oscillations associated with inertial effects.

### 2.2 Mesh sensitivity analysis

A sensitivity analysis is conducted to determine the optimal mesh density of the structural model. Four different hexahedral grids are constructed by increasing the number of elements in the thickness of the eye, thus decreasing the size of the elements. Mesh data are reported in Table 2. For each mesh, the zero-pressure configuration is computed, then the humors are pressurized at 15 mmHg. Finally, a static pressure is applied to the apex of the eye to study the apical deformation. In each case, the displacement of the apex at the instant of highest concavity is recorded as representative primary variable of the model. The error between two consecutive mesh sizes is calculated as

$$\text{error}\left[\%\right]=\frac{u_{\text{current}-\text{configuration}}-u_{\text{previous}-\text{configuration}}}{u_{\text{previous}-\text{configuration}}}\cdot 100$$ (5)

**TABLE 2:** Mesh data adopted in the mesh sensitivity analysis.

**Mesh analysis**		
Element in the thickness	Total number of elements	Total number of nodes
3	30236	20994
4	31991	22831
5	33746	24668
6	35501	26505

### 2.3 Fluid analysis and air puff simulation

**FIGURE 2:**
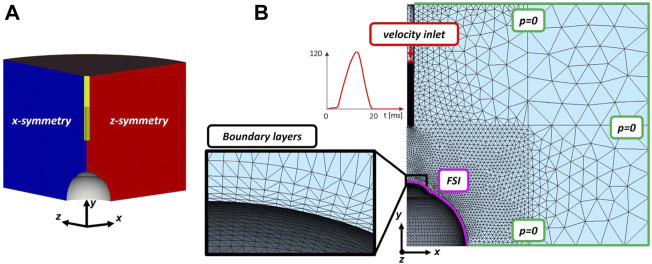
**(A)** 3D air domain with two symmetric planes; **(B)** surface mesh of the fluid domain with a zoom of the mesh on the five boundary layers at the FSI interface. Air velocity inlet in m/s (in red), pressure outlets (in green) and the FSI interface (in purple) are drawn.

The air domain (Figure 2) of the NCT test was set in 50 mm (3 times the radius of the eyeball) as it was observed to minimize boundary effects on the flow over the cornea. It presents two symmetric planes and a nozzle with an inlet Gaussian air-puff velocity (with a maximum of 120 $\frac{m}{s}$ during a period of 20 ms) at 11 mm from the corneal apex, that is the distance between the device and the eye as reported by the manufacturers (OCULUS, 2021). Zero pressure is imposed as an outlet boundary condition. The automatic volume mesher of the ICFD solver fills with 212,678 tetrahedral elements the input meshed surfaces. Five boundary layers are set at the FSI interface. The air domain is solved with an Arbitrary Lagrangian-Eulerian (ALE) kinematic approach. The air is modelled as an incompressible fluid whose density and dynamic viscosity are $\rho =1.25\cdot 10^{-6}\frac{g}{mm^{3}}$ , *μ* = 1.8 ⋅ 10^–8^ MPa⋅ms respectively. A turbulence model based on a variational multiscale approach is assumed. The fluid parameters are tested comparing the results of the computational fluid dynamic simulation (CFD) with the experimental results presented in Roberts et al. (Roberts et al., 2017). At the interface between the structure and the fluid a no-slip condition is adopted. NCT is simulated by a strongly coupled, 2-way and boundary fitted FSI. The NCT simulation consisted in a single step consisting in the inflation of the eye at a 15 mmHg IOP in the first 30 ms, followed by 10 ms of rest, to then apply the air puff between 40 and 60 ms.

### 2.4 Material sensitivity analysis

A sensitivity analysis is performed to identify the influence of the IOP and the material constants parameters. Five parameters governing the mechanical response of the eye are analysed: the IOP; the matrix stiffness parameter *C*_10−*c*_, the fibres stiffness parameter *k*_1_ and the fibres non-linearity parameter *k*_2_ of the cornea; and regarding the sclera the matrix stiffness parameter *C*_10−*s*_. A 2^5^ full factorial design which takes into account five different variables at two different levels (low and high) for a total of 32 different analysis is used. Data adopted in the analysis are reported in Table 3. The IOP and the material parameters are considered within a 50% of variation relative to the reference value. For each case, the air-puff FSI analysis as described in Section 2.3 is carried out and three biomarkers of the Corvis clinical test are examined:

• The deflection amplitude that is the displacement of the corneal apex at the instant of highest concavity and it gives information about the amplitude of the deformation of the cornea (Lopes et al., 2021) (Figure 6).
• The peak distance i.e., the distance between the two bending peaks on the cornea’s anterior surface at the instant of highest concavity. This markers gives information about the shape of the deformed cornea (Lopes et al., 2021) (Figure 6).
• The air pressure at the apex of the cornea at the instant of highest concavity.

**TABLE 3:** Low and high parameters tested in the material sensitivity analysis.

Material sensitivity analysis						
	Cornea		Sclera	Intraocular pressure		
Matrix stiffness	Fibres stiffness	Fibres non linearity	Matrix stiffness	
*C*_10−*c*_ [MPa]	*k*_1_ [MPa]	*k*_2_ [-]	*C*_10−*s*_ [MPa]	IOP [mmHg]
0.25–0.75	0.005–0.015	50–150	0.4–1.2	8–22

To study the main effects and the interaction effects of the input parameters on the responses, an ANOVA analysis is conducted by means of R-studio (RStudio Team, 2020).

### 2.5 Influence of the internal structures

A FSI analysis of the NCT without the internal structures of the eye (lens, zonule and vitreous membrane) is also conducted to evaluate their influence on three selected biomarkers. The variation of the IOP during the simulation, the deflection amplitude and the peak distance are compared with the same parameters of the simulation including the internal structures.

All the simulations described were performed using an Intel i9-10940X (3.30 GHz) with the finite-element solver LS-Dyna R 13.0 (LSTC, Livermore CA, United States) Dataset, 2021. The structural part is solved through a dynamic implicit structural solver, where as the air is modelled as an incompressible fluid and solved using the implicit ICFD solver ICF (2021).

## 3 Results

### 3.1 Mesh sensitivity analysis

**FIGURE 3:**
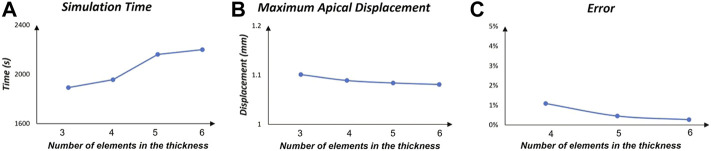
Mesh sensitivity analysis. The graphs are functions of the number of elements in the thickness. **(A)** Simulation time; **(B)** Displacement of the apex at the instant of maximum concavity; **(C)** Percentage error between consecutive mesh.

The results for the mesh sensitivity analysis are reported in Figure 3. As expected, the simulation time increases with the number of degrees of freedom in the model. The maximum apical displacement in the four cases ranges between 1.08 and 1.10 mm with the maximum error of 1.1% between the coarsest mesh and the mesh with four elements in the thickness. Based on the mesh convergence study, the mesh with five elements in the thickness is chosen for all the FSI simulations, which shows an error below 1%.

### 3.2 Fluid analysis

**FIGURE 4:**
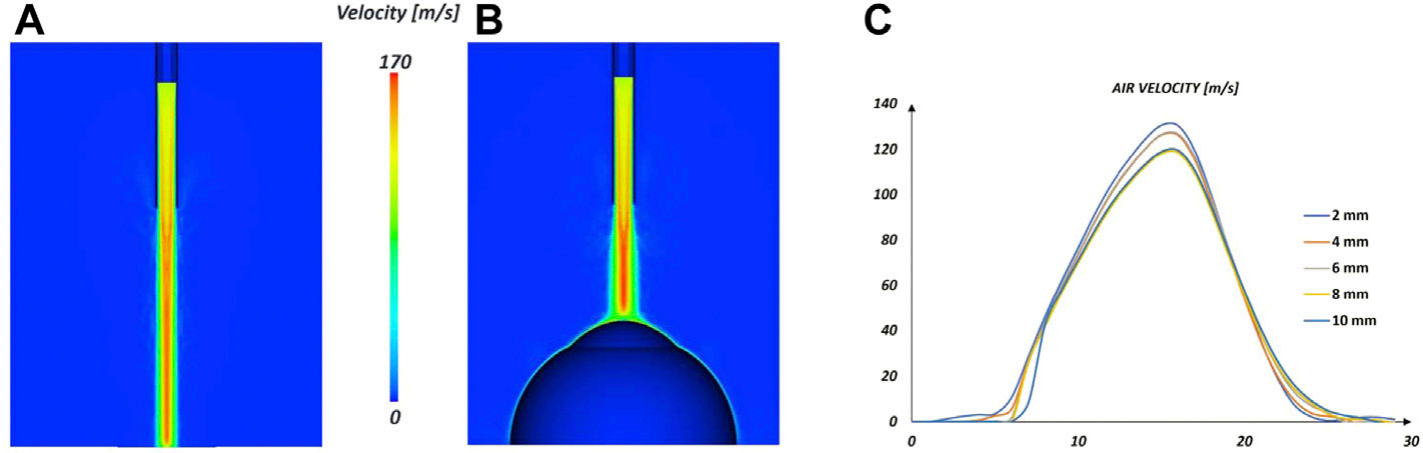
Fluid analysis: contour of the air peak velocity in the CFD simulation without the eye shape **(A)** and with the eye shape **(B)**. Centerline velocity distribution as a function of distance from the nozzle used to test the fluid parameters in the case without the eye shape.

In order to test the imposed boundary condition of the air-puff, a simulation of the air-puff with the fluid domain defined as described in paragraph 2.3 but without the eye has been carried out, to reproduce the results in Roberts et al. (Roberts et al., 2017). The fluid velocity during the air-puff at different distances from the nozzle along the centerline is recorded and reported in Figure 4. The range of the peak velocity (from 130.55 m/s at 2 mm to 119.9 m/s at 10 mm) from the numerical simulation was within the range reported in the experiments (from 133.57 m/s at 2 mm to 127.43 m/s at 10 mm) indicating that the time velocity profile used as boundary condition was appropriate to describe the air-puff.

### 3.3 FSI analysis

**FIGURE 5:**
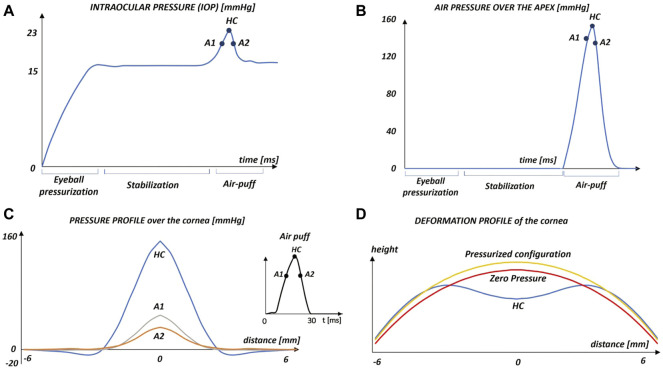
Results of the FSI simulation: **(A)** variation of the intraocular pressure (IOP) during the NCT simulation. The first applanation point (A1), highest concavity point (HC) and second applanation point (A2) are highlighted; **(B)** air pressure over the corneal anterior surface during the NCT simulation.**(C)** pressure profile over the cornea in three significant instants of the simulation as a function of the distance from the apex; **(D)** profile of the cornea in the zero pressure configuration, with an intraocular pressure of 15 mmHg and at the instant of highest concavity (HC).

One of the main objectives of the NCT is the evaluation of the IOP based on the Imbert-Fick principle, which states that in a flattened spherical body with an infinitely thin, dry, and elastic membrane wall, the internal pressure equals the force applied on the body divided by the applanation surface. Knowing the pressure exerted by the air puff and the applanation area derived from the images of Corvis ST Scheimpflug camera, the IOP is derived (Gonzalez Castro et al., 2016). Our simulation shows that the IOP varies during the NCT (Figure 5A). Since the eye is a closed volume filled with incompressible fluids, when the air jet deforms the corneal surface, the IOP increases reaching 22.5 mmHg (+50%).

On the air side, the pressure-time variation at the corneal apex during the air-puff is shown in Figure 5B. The maximum pressure in the apex, 152 mmHg, corresponds with the instant of maximum concavity of the cornea (*t* = 55 ms). The air pressure exerted over the corneal surface is reported in Figure 5C for A1, A2 and HC as a function of the distance from the apex. As expected, the maximum pressure is located at the apex of the cornea to then decrease. In the three cases, the pressure is zero for a distance from the apex higher than 5.5 mm, meaning that from this position, the eye does not perceive directly the air jet, the pressure component is negligible, and the flow follows the eye shape. In the instant of HC, there is a region where the air pressure is negative lending support to previous findings in the literature (Muench et al., 2019) (Ariza-Gracia et al., 2018) (Maklad et al., 2020a). Negative pressure occurs if the fluid reflects from the corneal surface in the opposite direction to the flow, causing the change of concavity of the cornea in the deformed state (Figure 5D, curve HC). The maximum pressure at A1 is higher than the maximum pressure at A2, due to the energy loss caused by the damping of the cornea and the deceleration of the air-jet during this phase of the test.

The deformation profile of the cornea is reported in Figure 5D where it is possible to appreciate the difference between the undeformed shape of the cornea’s central section in the zero-pressure configuration, in the pressurized configuration, and in the instant of highest concavity (HC). In the instant of HC, the deflection amplitude of the apex is 1.14 mm found to be within the range of clinical data (1.09 ± 0.10). (Roberts et al., 2017).

**FIGURE 6:**
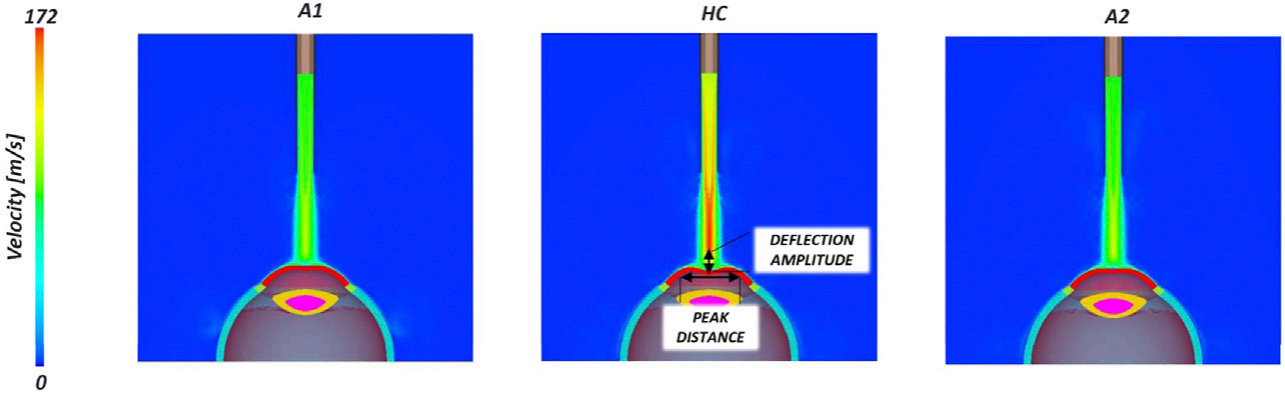
Air velocity contour in three-time points (A1, HC and A2) during the air-puff. In the instant of highest concavity two biomarkers (peak distance and deflection amplitude) are highlighted.

The contours of the air velocity in three significant points of the simulation are reported in Figure 6. In the instant of highest concavity the velocity (172 m/s) is higher than the inlet velocity due to an acceleration caused by the turbulent flow and the obstacle that the air meets, the eye structure.

**FIGURE 7:**
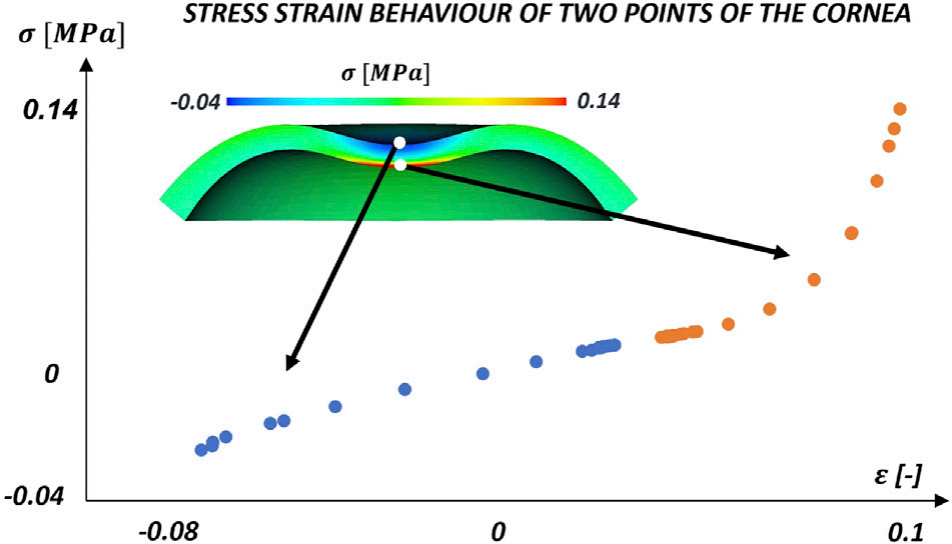
Evolution of normal stresses and strains at two locations (anterior and posterior) during the air puff. At the beginning of the air-puff both points are at a tension state due to the IOP. Then, during the air-puff, the anterior surface works in compression (blue) and the posterior in tension (red) with the contribution of the collagen fibres.

Physiologically, the cornea works in tension because of the IOP. However, during the air-puff, the cornea undergoes bending, therefore its anterior surface changes its state of tension to a compression state, whereas the posterior surface carries on working under tension as shown in the contour of the stress at the instant of HC (Figure 7). This means that the collagen fibres in the anterior surface do not contribute to load bearing during most of the duration of the air-puff, relying in this case on the mechanical properties of the matrix under compression. The evolution of the normal stress and strain values of two points of the apex (anterior and posterior surfaces) is plotted in Figure 7. As anticipated, the plot demonstrates the contribution of the collagen fibres when the cornea is subjected to tension because, after a first response of the matrix, a stiffer behaviour is reported. In compression (blue values), only the matrix contribution is present resulting in a more compliant response.

### 3.4 Material and IOP sensitivity analysis

The results of the ANOVA analysis for the biomarkers are reported in Table 4 and Table 5. For both the deflection amplitude and the peak distance, the IOP is the most important parameter followed by the matrix stiffness of the cornea, *C*_10−*c*_, with the other material parameters having little effect on the response. However, the effect size of the IOP is more than tenfold the size effect of *C*_10−*c*_. However, this could be associated with the relatively large range of IOP considered in the design of experiments. The results from the ANOVA also indicate a little interaction between the effects, being the *C*_10−*c*_⋅ IOP interaction and the *C*_10−*s*_⋅ IOP the most important, though very low with respect the effect of the singles (see Table 4.

**TABLE 4:** Analysis of variance (ANOVA) results on the deflection amplitude. Degrees of freedom (DF) and Effect size.

ANOVA for the deflection amplitude		
Source	DF	Effect
Linear	5	
*C*_10−*c*_	1	0.27
*k*_1_	1	0.03
*k*_2_	1	0.14
*C*_10−*s*_	1	0.14
IOP	1	4.01
2-way interactions	2	
*C*_10−*c*_⋅ IOP	1	0.03
*C*_10−*s*_⋅ IOP	1	0.03

**TABLE 5:** Analysis of variance (ANOVA) results on the peak distance biomarker. Degrees of freedom (DF) and Effect size.

ANOVA for the peak distance		
Source	DF	Effect
Linear	5	
*C*_10−*c*_	1	0.86
*k*_1_	1	0.17
*k*_2_	1	0.37
*C*_10−*s*_	1	0.15
IOP	1	17.05

**FIGURE 8:**
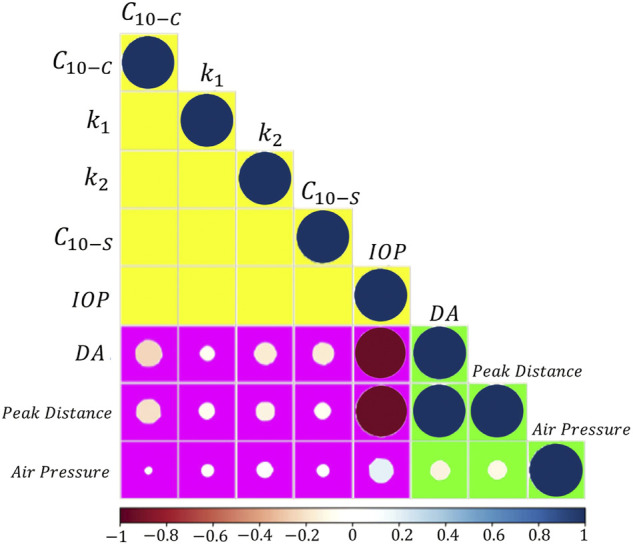
Pearson correlation matrix (full-level factorial design, 2^5^ = 32). Factors: matrix stiffness of the cornea (*C*_10−*C*_), fibres stiffness of the cornea (*k*_1_), fibres non linearity of the cornea (*k*_2_), matrix stiffness of the sclera (*C*_10−*s*_), intraocular pressure (IOP). Output parameters: deflection amplitude (DA), peak distance and air pressure over the cornea at the instant of maximum concavity. The colour of each circle depicts whether the linear correlation is direct (positive) or inverse (negative) (blueish palette, direct; reddish palette, inverse). The larger the circle diameter, the higher the correlation.

The Pearson correlation matrix in Figure 8 reports the results for the statistical analysis conducted. The dimension of each circle is related to the correlation between the factors of the design of experiment (*C*_10−*c*_, *k*_1_, *k*_2_, *C*_10−*s*_, IOP) and the dependent variables considered (deflection amplitude, peak distance, and air pressure over the apex). The colour of each circle tells whether the linear correlation is direct (blue) or inverse (red). The most important result that emerges from the data is the inverse correlation between the IOP and the deflection amplitude and the peak distance. As the IOP increases, the air pressure encounters a higher mechanical resistance, the deformation of the cornea is lower and, as a consequence, the deflection amplitude and the peak distance are lower. The figure also shows that the maximum air-pressure at the corneal apex is little influenced by the IOP and the material properties of the cornea and sclera.

**FIGURE 9:**
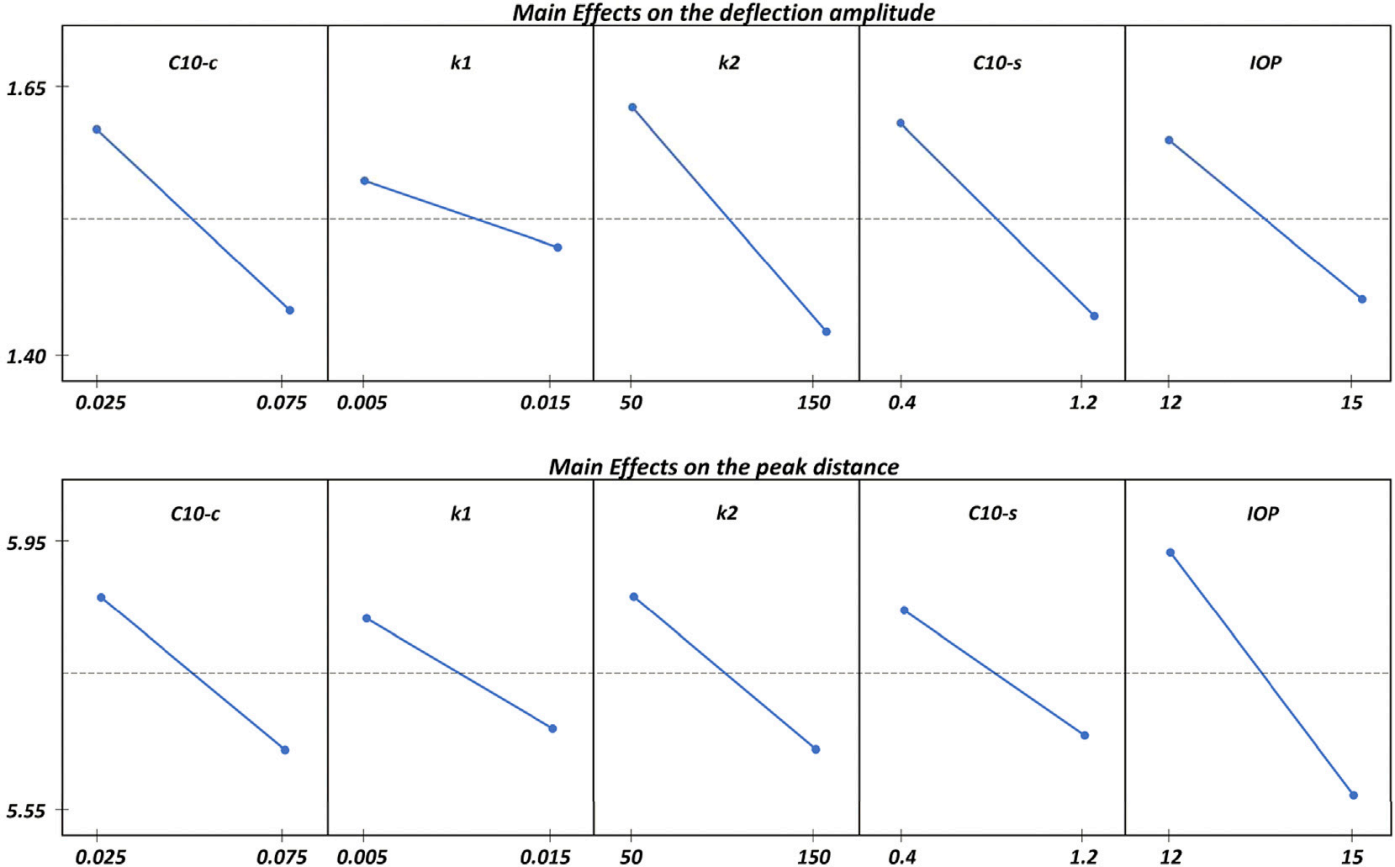
Main Effects plot of the analysed factors on the deflection amplitude and on the peak distance. All the parameters show an inverse influence on the results.

Given that these findings are based on two IOP levels of 8 and 22 mmHg, (values reached when the intraocular pressure is very low and when the patient presents glaucoma), a further analysis is conducted with the IOP ranging between 12 and 15 mmHg (the physiological range) and all the other parameters as described in Section 2.3. The main effect of the parameters on the deformation amplitude and the peak distance is shown in Figure 9. Each line depicts the difference in the mean response between the two levels of a factor, as long as each point represents the mean result for one level of a factor. The horizontal line indicate the average for all runs.

The results obtained for this reduced range of IOP are consistent with those shown in Figure 8, i.e., an inverse relationship with the corneal displacement and peak distance. However, within the physiological range of the IOP, the mechanical properties of the sclera have the same influence as the corneal matrix stiffness, whereas the anisotropic component of the cornea appears to plays a more important role on the corneal deformation against an air-puff.

### 3.5 Influence of the internal structures

The results for the analysis of the influence of the internal structures of the eye is reported in Table 6. The influence of the crystalline lens, the ciliary zonule and the vitreous membrane on the biomarkers was found to be always below 5%, meaning that they could be removed from the model improving the computational efficiency.

**TABLE 6:** Influence of the internal structure of the eye on the biomarkers.

Influence of the internal structures of the eye			
Biomarker	With internal structures	Without internal structures	Error (%)
IOP max	22.40 mmHg	23.25 mmHg	3.7
Deflection amplitude	1.402 mm	1.445 mm	3
Peak distance	5.54 mm	5.60 mm	1.16

## 4 Discussion

The deformation of the cornea during the NCT stems from the combination of different biomechanical factors; hence, to identify the influence of each factor, an *in silico* study is necessary. To the best of the authors knowledge, this paper is the first to propose a complete methodology capable to model the NCT using FSI considering an anisotropic formulation for the cornea and a complete fluid domain for the air. The results confirm the need of using FSI for the correct simulation of NTC as stated in Ariza Gracia et al. (Ariza-Gracia et al., 2018). The use of FSI, instead of only structural FEA, is key to capture the correct air pressure distribution over the cornea during the air-puff. The simulations show that the interaction of the air-puff with the deformable cornea causes a confinement of the flow in the proximity of the cornea (about 3.5 mm from the corneal apex) that increases the velocity of the jet-stream near the centerline with respect to what is seen in a free-flow condition (without obstacles) shown in Figure 4. This increment in the kinetic energy of the flow causes that the maximum pressure in the cornea reaches values of 152 mmHg, coincident with the moment when the air-puff reaches the maximum speed. Previous works estimated the pressure profile in the cornea as the dynamic pressure associated with the air-puff velocity profile (Ariza-Gracia et al., 2015) (Nguyen et al., 2019). However, doing so, the maximum pressure on the cornea maybe greatly underestimated as demonstrated in this study where the dynamic pressure associated with a velocity of 120 m/s results in 67 mmHg, almost half of the pressure obtained with the FSI analysis. The FSI proposed is 2-way and strongly coupled, thus iteratively the solid mechanics solver transfers the displacements of the eyeball to the fluid solver while the fluid solver transfers the air pressure to the solid mechanics solver. Thanks to this interaction, it is possible to appreciate the negative pressure in correspondence to the corneal peaks at the instant of maximum concavity, thus the dependence of the pressure on the biomechanical behaviour of the eyeball tissues.

The sensitivity analysis demonstrates the important role of the IOP on the corneal biomarkers during NTC. This effect has been found to be comparable to the effect of the material properties of the cornea and the sclera as reported in previous studies based on structural analysis (Ariza-Gracia et al., 2016) (Ariza-Gracia M.Á. et al., 2017). These results demonstrate the interplay between the mechanical properties of the eye and the IOP and the importance of taking into account this interaction when interpreting the results from a NTC test. Regarding the influence of the material properties, the sensitivity analysis confirms the results from (Ariza-Gracia et al., 2016) (Ariza-Gracia M.Á. et al., 2017) about the importance that corneal anisotropy has on the corneal biomarkers extracted during a NCT test, in particular for values of the IOP within the physiological range. This demonstrates the need of accounting for the anisotropic behaviour of the cornea in the models of the eye. At the instant of maximum concavity, the anterior surface of the corneal section works under compression, whereas the posterior surface works under tension. During tension, the collagen fibres are recruited which increases the corneal stiffness, and the load bearing capacity of the tissue. In fact, the material sensitivity analysis shows a contribution of the fibres parameters on the deformation amplitude and on the peak distance comparable to the extracellular matrix of the cornea.

Another remarkable result emerging from the material sensitivity analysis is the importance of the sclera: the stiffer the sclera, the lower the corneal deformation to the air-puff. Nguyen et al. (Nguyen et al., 2019) reported a similar trend in a computational-experimental work with *ex-vivo* human donor eyes. This indicates that an accurate model of the eye-ball to simulate the response to an air-puff should incorporate the sclera since otherwise the corneal displacements will be understimated. On the contrary, the sensitivity analysis shows a negligible effect of the remaining internal structures of the eye i.e., crystalline lens, ciliary zonule and vitreous membrane. This occurs in great part because of the lower stiffness of the components as happens with the zonule and the vitreous membrane which cause the crystalline lens to show a rigid body like motion during the NTC test (see the video in the support material section). Hence, depending on the aim of the simulation, the internal structure could be neglected. Last but not least, the simulation corroborates the findings from Montanino et al. (Montanino et al., 2018) (Montanino et al., 2019) and Ariza et at (Ariza-Gracia et al., 2018). that the humors need to be modelled as a fluid-like material with a specific density when simulating a NCT test. Since the eye-ball is a closed system and the humors behave as an incompressible fluid, the deformation of the cornea during the air-puff causes an increase in the IOP up to a 50% of the nominal value. Most experimental works regarding indentation (Ariza-Gracia M. et al., 2017) (Ortillés et al., 2017) or NTC (Nguyen et al., 2019) tests, impose a constant pressure in the eye-ball during the test by means of a column of water, which makes the humor to behave as an open system, contrary to what really happens during the actual test. The important influence that IOP has on the deformation of the cornea during the air-puff, makes that considering it as constant in the simulation causes an understimation of the corneal deformation, and therefore to overstimate the corneal stiffenss as a consequence.

The analysis is not absent of limitations. This study is based on an idealized geometry and the carried out sensitivity analysis did not consider the variability in the geometry which may significantly affect corneal deformation as shown in (Ariza-Gracia et al., 2015) (Ariza-Gracia et al., 2016) (Ariza-Gracia M.Á. et al., 2017). Further studies considering patient-specific geometries will be performed in order to quantify the variability in corneal biomarkers associated with the intrinsic geometric variability of the eye-ball. Also, two uniformly distributed families of fibers have been considered in the model (one in the nasal-temporal direction and another in the superior-inferior direction) ignoring the collagen fiber dispersion present in the human cornea (Pandolfi and Holzapfel, 2008; Meek and Boote, 2009). This issue will be accounted for in future developments. Moreover, although the analysis reveals that the removal of the internal structures produces a < 5% change in the data of interest, the influence of the ciliary body in the eye-ball response to an air-puff should be further investigated. Lastly, a future line of the work could be the simulation of the whole eye movement during the air puff incorporating in the model also the posterior half of the eye globe.

## 5 Conclusion

This paper has highlighted the importance of conducting an FSI simulation to model the NCT. Moreover, it has underlined that the humors should be modeled as a fluid rather than a constant homogeneous pressure. As the results pointed out, the corneal deformation is strongly influenced by the mechanical properties of the cornea and the sclera, therefore a correct formulation for both parts must be included in the simulation. In particular, an anisotropic material is mandatory for the cornea.The simulation proposed can be used as starting point for further analysis regarding the study of eye biomechanics based on clinical data. Future works in modeling the non-contact tonometry should be addressed to patient-specific simulations with the correct corneal geometry and material properties. Last but not least, since for a given geoemetry i.e., a patient specific geometry, the biomarkers derived from the NCT depend mainly on the material parameters of the cornea and sclera, it would be possible to apply the inverse finite element method to find the mechanical properties of these tissues for *in vivo* characterization.

## Data Availability

The original contributions presented in the study are included in the article/Supplementary Material, further inquiries can be directed to the corresponding author.
